# 

**Published:** 1871-02

**Authors:** 


BOOKS RECEIVED.

*General Surgical Pathology and Therapeutics, in Fifty Lectures. A Text Book for Students and Physicians.* By Dr. Theodor Billroth, Professor of Surgery in Vienna. Translated from the Fourth German Edition, with the special permission of the author, by Charles E. Hackley, A.M., M.D., Surgeon to the New York Eye and Ear Infirmary, etc. New York: D. Appleton & Co., 90, 92 and 94 Grand street. 1871. Pp. 676.

*On Diseases of the Spine and Nerves.* By Charles Bland Radcliffe, M.D., F. R. C. P., Lond., etc.; John Netten Radcliffe, Medical Sup't Hospital, etc.; J. Warburton Begbie, M.D., F. R. C. P., Edin., etc.; Francis Edmund Ainstie, M.D., F. R. C. P., etc., etc.; and John Russell Reynolds, M.D., F. R. S., etc., etc. Philadelphia: Henry C. Lea. 1871. Pp. 196.

*On the Wasting Diseases of Infants and Children.* By Eustace Smith, M.D., Lond., M. R. C. P., etc., etc. Second American, from the Second Revised and Enlarged English Edition. Philadelphia: Henry C. Lea. 1871. Pp. 266.

*Body and Mind: An Inquiry into their Connection and Mutual Influence, Specially in Reference to Mental Disorders;* being the Gulstonian Lectures for 1870, delivered before the Royal College of Physicians; with an Appendix. By Henry Maudsley, M.D., Lond., etc., etc. New York: D. Appleton & Co. 1871. Pp. 155.

*Bartholow on Spermatorrhœa.* New Edition.

*Satan in Society.* By a Physician.

"Here are a few of the unpleasant'st words
That ever blotted paper." *Shakspeare.*

Cincinnati and New York: C. F. Vent. Chicago: J. S. Goodman & Co. 1871. Pp. 412.

[This book is noticed by a well known correspondent of the Journal, in terms which, in the main, so fully accord with our own views, that we publish his article (p. 108) in preference to one we had partially prepared. The book may gain something of notoriety by the space allotted to it, but the class it represents is getting so unblushing in its effrontery, that we had rather let our correspondent rebuke it in the mild terms he has chosen, than to employ the harsher adjectives we had ourselves selected. It is the culminating atrocity of the press.]

*Circular No.* 3. *Approved Plans and Specifications for Post Hospitals.*

*Circular No.* 4. *Report on Barracks and Hospitals, with Descriptions of Military Posts.* War Department, U. S. A., Surgeon General's Office, Washington, Nov. 23d and Dec. 5, 1870.

Circular No. 4 is a quarto volume of 494 pages, giving a full account, with detailed descriptions, plats, diagrams, etc., of all the Government Barracks and Hospitals.

Circular No. 3 is in the same form, but gives only about two pages of description and five lithographic plans of hospitals, ventilating arrangements, etc. The two constitute a portly volume, from which we shall hereafter draw largely in our efforts to secure "modern improvements" in the hospitals hereafter to be constructed, wherever the Journal finds readers. It may astonish some of our readers to find out that there has recently been constructed in this city one of the old-time castellated palaces of *ochlesis*, putrescence and death, and this under the very droppings of the Medical Reform School. Yet others of the same antediluvian type are in contemplation.

We were about to say that it would be better for the unfortunate sick to be exposed as in olden time upon the waysides, or given a *clean* air out upon the open prairie.

Thanks to Surgeon General Barnes and his corps of earnest assistants, the Government for once is far in advance of the people, and even of a large number of the fossils of the profession.

PAMPHLETS RECEIVED.

*Histological Contribution.* By H. G. Pifford, M.D., Surgeon to New York Dispensary for Diseases of the Skin.

*Blood-letting as a Therapeutic Resource in Obstetric Medicine.* By Fordyce Barker, M.D., etc., etc.

*The New York Observer Year Book and Almanac for* 1871. Sidney E. Morse, Jr. & Co., 37 Park Row, New York City.

A bulky octavo of 200 pages, price $1, but given gratuitously to all advance paying subscribers of that time-honored and excellent religious paper, the *New York Observer.* It contains a vast amount of statistical and current information, and reprints entire the first Directory of New York, published in 1786.

*Scribner's Monthly.* An Illustrated Magazine for the People. Conducted by J. G. Holland (Jan., 1871): published by Scribner & Co., New York. $3.00 a year.

*The Young Pilot.* An Original Monthly Magazine for young people in their teens. Jan., 1871. $1.00 per annum. Chicago: The Young Pilot Publishing Co.

*The Relations of the Medical Profession to Modern Education.* By Edward S. Dunster, M.D. New York: D. Appleton & Company.

*Transactions of the Twentieth Anniversary of the Illinois State Medical Society*, held in Dixon, May 17, 18, 1870. Robert Fergus' Sons, Printers. Pp. 141.

*Syphilis of the Nervous System.* A Clinical Study, chiefly in regard to Diagnosis and Treatment. Founded on the cases of Prof. Wm. H. Van Buren, M.D., and those of the author. By E. L. Keyes, M.D., etc. New York: D. Appleton & Co. Pp. 44.

*Retention of Urine Depending on Stricture.* By Alexander W. Stein, M.D., etc., etc. Pp. 16.

*Sixth Annual Report* of the Illinois Institution for the Education of Feeble Minded Children, located at Jacksonville, to the Governor of Illinois. Dec. 1870. "Comfort the feeble-minded." Pp. 52.

*Quarterly Summary* of the Transactions of the College of Physicians and Surgeons of Philadelphia, from Oct. 6th, 1869, to May 18th, 1870, inclusive.

*Proceedings of the Convention* for the Reorganization of the Medical Society of the State of California, and of the first Annual Meeting, etc., etc. Incorporated Nov. 1, 1870. Pp. 41.

*Annual Report* of the Board of Trustees of the Hospital for the Insane of the State of Wisconsin. Sept. 30, 1870. Pp. 100. From the Superintendent.

